# Associations of Handgrip Strength with Bone Health and Mental Health in Postmenopausal Women: A Cross-Sectional Study

**DOI:** 10.3390/medicina62010055

**Published:** 2025-12-28

**Authors:** Marin Mornar, Josko Bozic, Nikola Pavlovic, Josip Vrdoljak, Marko Kumric, Tina Vilovic, Tina Ticinovic Kurir, Marko Grahovac, Marino Vilovic

**Affiliations:** 1 Department of Endocrinology, Diabetes and Metabolic Diseases, University Hospital of Split, Spinciceva 1, 21000 Split, Croatia; marin.mornar@mefst.hr (M.M.); tticinov@mefst.hr (T.T.K.); marko.grahovac@mefst.hr (M.G.); 2 Department of Pathophysiology, University of Split School of Medicine, Soltanska 2A, 21000 Split, Croatia; josko.bozic@mefst.hr (J.B.); nikola.pavlovic@mefst.hr (N.P.); josip.vrdoljak@mefst.hr (J.V.); marko.kumric@mefst.hr (M.K.); 3 Laboratory for Cardiometabolic Research, University of Split School of Medicine, Soltanska 2A, 21000 Split, Croatia; 4 Department of Family Medicine, University of Split School of Medicine, Soltanska 2A, 21000 Split, Croatia; tina.vilovic@mefst.hr; 5 Department of Family Medicine, Split-Dalmatia Health Center, Kavanjinova 2, 21000 Split, Croatia

**Keywords:** hand strength, postmenopause, bone density, dual-energy X-ray absorptiometry, mental health, resilience, self-control, cross-sectional studies

## Abstract

*Background and Objectives*: Handgrip strength (HGS) is a simple marker of muscular fitness that has been linked to adverse outcomes in older adults, while menopause is accompanied by skeletal deterioration and increased psychological vulnerability. Resilience and self-regulation may be associated with lower levels of these risks, but their relationship with bone microarchitecture has not been clarified. We aimed to examine the associations between HGS and trabecular bone score (TBS), bone mineral density (BMD), mental health, resilience, and self-regulation in postmenopausal women. *Materials and Methods*: In this study, 200 postmenopausal women were recruited. HGS was assessed with a dynamometer, BMD at the lumbar spine, total hip and femoral neck by DXA, and lumbar TBS was derived from spine images. Psychological distress was measured with the DASS-21, resilience with the Brief Resilience Scale (BRS), and self-regulation with the Short Self-Regulation Questionnaire (SSRQ). *Results*: TBS was significantly higher in women with higher HGS (*p* < 0.001). Higher HGS was also associated with lower anxiety and depression scores (*p* = 0.011 and *p* = 0.013), fewer self-reported mental health disorders, and greater resilience (*p* < 0.001) and self-regulation (*p* = 0.004). Resilience and self-regulation were inversely related to all DASS-21 subscales (all *p* < 0.001), and HGS correlated positively with BRS (*p* < 0.001) and SSRQ (*p* < 0.001). TBS correlated modestly with both BRS (*p* = 0.003) and HGS (*p* < 0.001). In multiple linear regression, both BRS (β = 0.018, *p* = 0.013) and HGS (β = 0.003, *p* = 0.006) remained independently associated with TBS after adjustment for age, BMI, menopause duration, and SSRQ. *Conclusions*: In postmenopausal women, higher handgrip strength is associated to better trabecular bone microarchitecture and a more favorable psychological profile. Incorporating HGS and brief psychosocial assessment alongside TBS may enrich fracture risk stratification and support more integrated musculoskeletal and mental health care.

## 1. Introduction

Handgrip strength (HGS) is widely recognised as a simple and reliable marker of overall muscle strength and functional capacity, and is increasingly used as a practical indicator of general health in older adults [1,2]. In women, muscle strength commonly declines with age, and this process may accelerate around the menopausal transition due to endocrine and metabolic changes, particularly the reduction in estrogen levels [3,4]. Reduced HGS is associated not only with impaired physical function, but also with adverse health outcomes and poorer quality of life, including limitations in mobility, self-care, and daily activities [5,6,7]. Consequently, HGS represents an accessible clinical metric that may help identify postmenopausal women at increased risk of functional decline.

In parallel, bone metabolism undergoes profound changes during and after menopause. Estrogen deficiency triggers an imbalance characterized by increased bone resorption and altered bone formation, leading to accelerated bone loss and heightened risk of osteoporosis and fractures [8,9]. Studies demonstrate that bone turnover markers remain elevated for decades postmenopause, underscoring the sustained impact of hormonal changes on bone remodeling and mineral density [10]. For this reason, assessing not only bone mineral density but also parameters reflecting bone microarchitecture is especially relevant in postmenopausal women to anticipate and mitigate skeletal fragility.

The interplay between muscle strength, particularly handgrip strength, and bone metabolism is biologically plausible, as muscular forces exerted on bones serve as critical mechanical stimuli for bone remodeling and strength preservation [11]. Declining muscle strength may therefore contribute to deteriorations in bone health, although the precise mechanisms and clinical implications in postmenopausal women require further elucidation [12,13].

Beyond musculoskeletal health, menopause is also associated with increased vulnerability to psychological symptoms, including anxiety, depression, and mood fluctuations that can markedly impair well-being [14,15,16]. Psychosocial factors such as resilience and self-regulation have emerged as important protective constructs in mental health during this period [17]. Resilience encompasses optimism, emotional stability, self-esteem, and adaptive emotion regulation, and has been empirically associated to better mental well-being among menopausal women [18]. Moreover, recent evidence suggests that handgrip strength in older adults correlates with mental health outcomes, including stress levels and depressive symptoms, indicating psychophysiological interconnections [19,20].

The concept of self-regulation, the capacity to modulate emotions, thoughts, and behaviors, adds a critical dimension to understanding how women navigate the biopsychosocial challenges of menopause [18]. Effective self-regulation may be associated with higheer resilience and lower mental health symptoms, potentially influencing physical health markers such as muscle strength and bone metabolism through neuroendocrine and behavioral pathways [21].

Given that menopause affects physical and psychological health simultaneously, investigating these domains together may provide a more comprehensive understanding of vulnerability and adaptation in postmenopausal women. Therefore, this study aims to investigate the intricate relationships among handgrip strength, bone metabolism, mental health, resilience, and self-regulation in postmenopausal women, given the complicated effects of menopause on both physical and mental health. Maintaining musculoskeletal integrity and psychological well-being in this susceptible group may be better understood by bridging these interconnected realms.

## 2. Materials and Methods

### 2.1. Study Sample

This cross-sectional study was conducted at the Department of Endocrinology, Diabetes, and Metabolic Diseases, University Hospital of Split in the period between April 2024 and June 2025. It included 200 postmenopausal women (mean age 67.5 ± 7.1 years), recruited via convenience sampling during regular clinical examinations. Sample size was determined using G*Power 3.1. For the primary outcome (difference in lumbar spine TBS between HGS groups), assuming α = 0.05, power = 0.90, and a moderate effect size (Cohen’s d = 0.50), an independent-samples t-test with a 1:1 allocation required 172 participants (86 per group). To ensure an adequate final sample, we planned to recruit more than 180 participants and ultimately enrolled 200 women. For secondary psychosocial outcomes (resilience, self-regulation, and DASS subscale scores), a bivariate correlation with HGS (α = 0.05, power = 0.90, r = 0.25) required 164 participants, indicating that the final sample also provided adequate power for these associations.

Women were eligible if they were postmenopausal, defined as ≥12 consecutive months of amenorrhea or prior bilateral oophorectomy. Exclusion criteria were as follows: BMI > 37 kg/m^2^, because TBS derived from lumbar spine DXA is recommended only within the validated BMI range (approximately 15–37 kg/m^2^) due to potential soft-tissue-related artifacts and reduced measurement validity outside this range [22]; presence of bone metastases or other malignant skeletal involvement, due to their profound effects on bone structure and DXA-derived parameters; and inability to complete the questionnaires (e.g., insufficient comprehension or cognitive/functional limitations preventing reliable self-report).

Participants were stratified into two groups according to handgrip strength (HGS): lower HGS (mean 20.1 ± 3.8 kg, *N* = 101) and higher HGS (mean 28.7 ± 3.2 kg, *N* = 99). However, HGS was analysed primarily as a continuous variable. For interpretability, we performed low vs. high HGS group comparisons as well, using the sample median cut-off (24.7 kg). This reflects within-sample distribution and is not intended as a clinical classification. Commonly used thresholds for low grip strength in older women include <16 kg [23]. Baseline anthropometric parameters (body weight, height, BMI, waist, hip, and thigh circumferences) and clinical data (menopause duration, smoking status, presence of bone-affecting non-communicable diseases, and bone loss therapies) were collected. All participants provided informed consent, and the study was conducted in accordance with the Declaration of Helsinki and approved by the Ethics Committee of the University of Split School of Medicine (No: 2181-198-03-04-23-0083).

#### Study Procedures

All participants were evaluated during routine outpatient visits at the University Hospital of Split. Data collection was performed on working days (Monday–Friday) in the morning hours during regular check-ups (approximately 08:00–12:00) to standardize assessment conditions. Participants underwent a standardized visit that included review of medical history and current therapies, anthropometric measurements, completion of the psychosocial questionnaires and functional and bone assessment (handgrip strength and DXA scanning). Short rest periods were provided between tests, and all measurements were performed by trained personnel using the same equipment and standard protocols throughout the study.

### 2.2. Handgrip Strength and Bone Health Evaluation

Handgrip strength (HGS) was assessed using a calibrated Jamar^®^ Smart hydraulic hand dynamometer (Performance Health Supply, Inc., Cedarburg, WI, USA), in accordance with standardized protocols. Measurements were performed with the participant seated in an upright position, the shoulder adducted and neutrally rotated, the elbow flexed at 90°, the forearm in a neutral position, and the wrist between 0° and 30° dorsiflexion [24].

Each participant performed three maximal voluntary contractions with the dominant hand, with a brief rest period between trials to minimize fatigue. The highest value (in kilograms) obtained from the three attempts was used for analysis. Participants were classified into lower and higher HGS groups using the sample median as the cut-off.

Bone mineral density (BMD) measurements of lumbar spine (L1–L4), total hip, and femoral neck were obtained by dual-energy X-ray absorptiometry (DXA) using a QDR 4500 C Bone Densitometer (Hologic, Marlborough, MA, USA). The device was calibrated daily with an anatomical spine phantom. BMD results were expressed as g/cm^2^, and corresponding T-scores and Z-scores were automatically computed based on normative reference data. Trabecular Bone Score (TBS) was calculated from DXA lumbar spine images using TBS iNsight software (version 3.0.2.0, Medimaps Group SA, Geneva, Switzerland) to assess bone microarchitecture quality. TBS values were classified as normal (≥1.350), partially degraded (1.200–1.350), or degraded (≤1.200), according to the proposed guidelines for the postmenopausal women population [25].

### 2.3. Psychosocial and Mental Health Questionnaires

Mental health and resilience were evaluated using the Depression, Anxiety, and Stress Scale (DASS-21), the Brief Resilience Scale (BRS), and the Short Self-Regulation Questionnaire (SSRQ). These validated instruments measured symptoms of depression, anxiety, stress, resilience capacity, and self-regulatory abilities that may influence health outcomes. In addition to validated questionnaires, we captured a self-reported history of mental health problems using a single questionnaire item. Participants were coded as ‘yes’ if they reported either (a) a professionally confirmed mental health diagnosis by a licensed professional or (b) self-perceived but undiagnosed mental health problems. This variable was used as a secondary descriptive indicator, whereas mental health symptom burden was primarily evaluated using the validated DASS-21 subscales.

Psychological distress was assessed with the DASS-21 using the official Croatian translation. Each subscale (Depression, Anxiety, Stress) comprises 7 items scored 0–3, and subscale totals were multiplied by 2 to align with the full DASS-42 norms and severity bands. Depression severity was categorized as normal (0–9), mild (10–13), moderate (14–20), severe (21–27), and extremely severe (≥28). Anxiety severity was categorized as normal (0–7), mild (8–9), moderate (10–14), severe (15–19), and extremely severe (≥20). Stress severity was categorized as normal (0–14), mild (15–18), moderate (19–25), severe (26–33), and extremely severe (≥34). The Croatian DASS-21 has demonstrated excellent internal consistency and construct validity in adult samples, including clinical validation, supporting its use in Croatian populations [26,27]. In our sample, Cronbach’s alpha was 0.90 for the Depression subscale, 0.85 for Anxiety, and 0.83 for Stress, indicating good to excellent internal consistency.

Resilience was assessed using the Brief Resilience Scale (BRS), which conceptualizes resilience as the capacity to “bounce back” following stress. The BRS comprises 6 items rated on a 5-point Likert scale (“strongly disagree” to “strongly agree”). Each item is scored 1–5, and the total score is the arithmetic mean of all six items (range 1.00–5.00), with higher values indicating greater resilience. Participants were classified as having low resilience (1.00–2.99), normal resilience (3.00–4.30), or high resilience (4.31–5.00) [28]. The BRS is widely used and has shown good psychometric properties across languages, including a Croatian adaptation with Cronbach’s α = 0.82 [29]. In our sample, internal consistency for the BRS was Cronbach’s α = 0.89.

Self-regulatory capacity was measured using the Short Self-Regulation Questionnaire (SSRQ), a 31-item instrument that assesses goal setting, self-monitoring, and self-adjustment processes underlying self-regulation [30]. Each item is rated on a 5-point Likert scale from 1 (“strongly disagree”) to 5 (“strongly agree”), with higher scores reflecting greater self-regulatory ability. Since no official Croatian version of the SSRQ is available, we employed a rigorous back-translation procedure by the two bilingual experts independently. Discrepancies were resolved through consensus to ensure semantic, conceptual, and cultural equivalence of items. In our sample, the SSRQ demonstrated excellent internal consistency (Cronbach’s α = 0.93), consistent with prior validation studies reporting reliability coefficients above 0.85 in diverse populations [30,31].

### 2.4. Statistical Analysis

Continuous variables were expressed as mean ± standard deviation or median with interquartile range, depending on distribution normality assessed by the D’Agostino–Pearson test. Homogeneity of variances was assessed using the F-test. For normally distributed variables, an independent-samples t-test was used when variances were homogeneous, and when variances were unequal (F-test *p* < 0.05), the Welch *t*-test was applied. For non-normally distributed continuous variables, the Mann–Whitney U test was used. Categorical variables (e.g., smoking status, bone status categories, TBS categories, fragility fractures, DASS-21 severity categories, resilience categories, and SSRQ tertiles) were compared using chi-squared tests. Effect sizes were reported to aid interpretation. For between-group comparisons of key continuous variables, Hedges’ g with 95% confidence intervals was calculated, with effect magnitude interpreted as trivial (<0.20), small (0.20–0.59), moderate (0.60–1.19), large (1.20–1.99), very large (2.00–3.99), and extremely large (≥4.00), using absolute values.

Correlations between HGS, bone parameters (BMD, TBS, FRAX estimates), anthropometric measures, and psychosocial scores (DASS-21, BRS, SSRQ) were examined using Pearson or Spearman correlation coefficients, according to variable distribution and scale. For correlation analyses, the strength of correlations was interpreted as negligible (0.00–0.10), weak (0.10–0.39), moderate (0.40–0.69), strong (0.70–0.89), and very strong (0.90–1.00). To examine variables independently associated with bone microarchitecture, we constructed a prespecified multiple linear regression model with TBS as the dependent variable. Predictors were selected based on established literature and biological plausibility as potential confounders or key variables related to muscle strength, psychosocial functioning, and bone health. This approach was used to control confounding rather than relying on data-driven variable selection Model assumptions were assessed using standard diagnostic procedures (inspection of residuals and evaluation of multicollinearity). Finally, as a sensitivity analysis to complement unadjusted low vs. high HGS comparisons, we performed analysis of covariance (ANCOVA) comparing TBS and key psychosocial outcomes between HGS groups, with handgrip strength group as the main factor and age, BMI, and menopause duration entered as covariates. Adjusted group differences were reported as regression coefficients (β) with 95% confidence intervals. Statistical significance was set at *p* < 0.05 (two-tailed), while all analyses were performed using MedCalc statistical software (version 19.1.2). Effect size calculations were additionally performed using the Python programming language (version 3.11) with the SciPy and NumPy libraries. Given the number of outcomes examined, *p*-values for secondary analyses were interpreted descriptively. No formal multiplicity correction was applied, and these findings should be considered exploratory.

## 3. Results

The study included 200 postmenopausal women (mean age 67.5 ± 7.1 years). Participants with higher handgrip strength (HGS) were significantly younger (65.9 ± 6.9 vs. 69.1 ± 6.9 years; *p* = 0.002) and taller (167.2 ± 6.4 vs. 164.8 ± 6.3 cm; *p* = 0.009) compared to those with lower HGS, whereas body weight, BMI, and body circumferences did not differ significantly between groups. The prevalence of active smoking showed a non-significant trend toward higher rates in the high-HGS group (28.3 vs. 16.8%; *p* = 0.053). All baseline parameters can be seen in Table 1.

**Figure 1:**
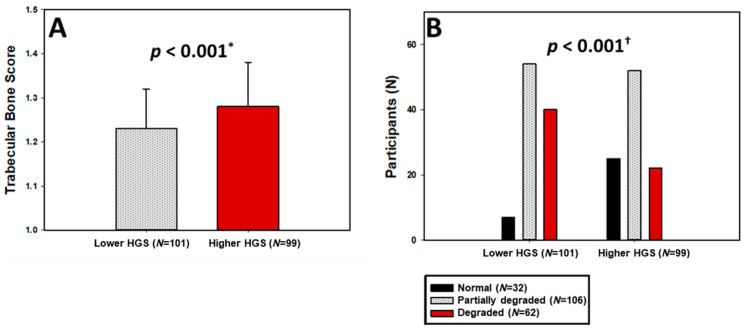
Trabecular bone score levels (**A**) and categories of bone microarchitecture degradation (**B**) stratified by HGS groups. HGS—Handgrip Strength; * *t*-test for independent samples; ^†^ chi-squared test.

Differences in bone mineral density (BMD) between the two HGS groups were not statistically significant across lumbar spine, total hip, or femoral neck regions (all *p* > 0.05; Table 2). However, lumbar spine Z-scores differed modestly between groups (*p* = 0.025), while hip and femoral neck Z-scores did not. Moreover, trabecular bone score (TBS) values were significantly higher among women with higher HGS (1.28 ± 0.10 vs. 1.23 ± 0.09; *p* < 0.001; Hedges’ g = 0.5 (0.22, 0.78); Figure 1A), accompanied by more favorable TBS T- and Z-scores (*p* < 0.001 and *p* = 0.047, respectively).

When classified according to bone microarchitecture, 78.1% of participants with higher HGS had normal TBS compared to only 21.9% in the lower HGS group, whereas degraded microarchitecture was almost twice as prevalent among women with low HGS (39.6% vs. 22.2%; *p* < 0.001; Figure 1B).

Consistent with these findings, TBS-adjusted FRAX estimates indicated a significantly lower 10-year probability of major osteoporotic fracture (10.0 (7.7–15.0) vs. 13.0 (9.9–17.2) %; *p* = 0.015) and hip fracture (1.5 (0.52–3.37) vs. 2.4 (1.1–4.1) %; *p* = 0.017) among women with higher HGS. The prevalence of fragility fractures was significantly lower in the high-HGS group (9.1% vs. 25.7%; *p* = 0.002).

Participants with higher HGS had significantly lower anxiety (DASS-A) and depression (DASS-D) scores (*p* = 0.011 and *p* = 0.013, respectively; Table 3). Also, effect sizes were small-to-moderate, with Hedges’ g = −0.54 (−0.83, −0.26) for anxiety, −0.46 (−0.74, −0.18) for depression, and −0.31 (−0.59, −0.03) for stress, with the higher HGS group showing lower scores. Normal anxiety levels were observed in 87.8% of the high-HGS group compared with 61.4% of those with lower HGS, whereas severe or extremely severe anxiety was reported by 13.9% of the latter and only 1.0% of the former (*p* < 0.001). A similar pattern emerged for depression, where mild-to-moderate or severe symptoms occurred in 18.8% of the low-HGS versus 5.1% of the high-HGS participants (*p* = 0.009). Stress levels did not differ significantly by group in terms of raw scores (*p* = 0.136), although a greater proportion of women with higher HGS fell within the normal stress category (90.9 vs. 76.2%; *p* = 0.019). Finally, a self-reported history of mental health problems (clinically diagnosed or self-perceived) was also more common in the lower HGS group (25.7% vs. 14.1%; *p* = 0.041). Detailed analysis of mental health parameters can be seen in Table 3.

**Figure 2:**
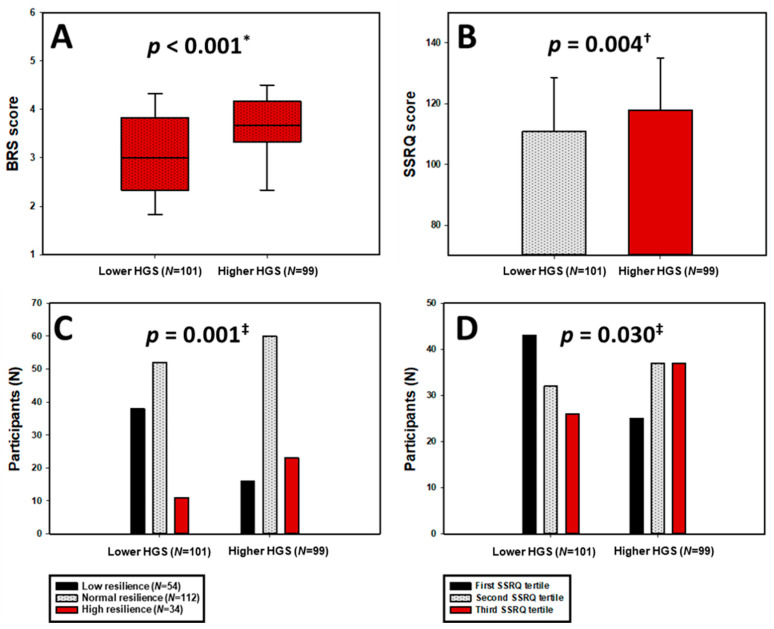
Distribution of BRS scores (**A**) and SSRQ scores (**B**), as well as BRS categories (**C**) and SSRQ tertiles (**D**), stratified by HGS groups. HGS—Handgrip Strength; BRS—Brief Resilience Scale; SSRQ—Short Self-Regulation Questionnaire. * Mann–Whitney U test. ^†^ *t*-test for independent samples. ^‡^ chi-squared test.

Furthermore, as shown in Figure 2A,B, participants with higher HGS demonstrated significantly greater resilience levels (median BRS = 3.67 (3.33–4.17) vs. 3.00 (2.33–3.83); *p* < 0.001; Hedges’ g = 0.67 (0.39, 0.96)) and self-regulatory capacity (mean SSRQ = 117.99 ± 17.07 vs. 110.86 ± 17.73; *p* = 0.004; Hedges’ g = 0.4 (0.12, 0.68)).

Categorically, as seen in Figure 2C,D, high resilience (BRS ≥ 4.31) was observed in 23.2% of women with higher HGS compared to 10.9% in the low-HGS group, whereas low resilience was more than twice as prevalent among those with lower HGS (37.6 vs. 16.2%; *p* = 0.001). Likewise, the distribution across SSRQ tertiles indicated a significant shift toward better self-regulation among the high-HGS group (*p* = 0.030).

Across the total sample, HGS correlated inversely with age (r = −0.227; *p* = 0.001), while no significant correlations were found with BMD values (Table 4). HGS also showed moderate negative correlations with anxiety (r = −0.255; *p* < 0.001), depression (r = −0.248; *p* < 0.001), and stress (r = −0.200; *p* = 0.005). Both resilience (BRS) and self-regulation (SSRQ) scores were significantly inversely associated with all DASS-21 subscales (*p* < 0.001). Also, HGS showed significant positive correlation with BRS score (r = 0.304; *p* < 0.001) and SSRQ score (r = 0.267; *p* < 0.001).

Finally, TBS correlated modestly yet significantly with both BRS (r = 0.206; *p* = 0.003) and HGS (r = 0.249; *p* < 0.001) (Table 4). Furthermore, multiple linear regression model showed that both BRS score (β = 0.018, SE = 0.007, t-value = 2.50, *p* = 0.013) and handgrip strength (β = 0.003, SE = 0.001, t-value = 2.79, *p* = 0.006) retained significant association with TBS, set as the dependent variable, when computed alongside baseline characteristics (age, BMI, and menopause duration) and SSRQ score.

Also, in sensitivity analyses adjusting for age, BMI, and menopause duration, the differences between HGS groups remained consistent with the unadjusted analyses. Higher HGS was associated with higher TBS (β = 0.041, 95% CI 0.015–0.066, *p* = 0.002), higher resilience (β = 0.558, *p* < 0.001) and self-regulation (β = 6.55, *p* = 0.010), and lower psychological distress (DASS-A: β = −3.03, *p* < 0.001; DASS-D: β = −2.07, *p* = 0.003; DASS-S: β = −2.29, *p* = 0.034).

## 4. Discussion

In this cross-sectional study of postmenopausal women, higher handgrip strength was significantly associated with superior trabecular bone score values and better psychological well-being characterized by lower anxiety and depression, greater resilience, and stronger self-regulatory capacity. Moreover, multivariate regression identified both HGS and resilience as independent correlates of bone microarchitecture quality, suggesting that the integrity of skeletal and psychosocial domains may share common biological and behavioral pathways.

We could argue that the observed association between muscle strength and bone microarchitecture is consistent with mechanostat theory, which posits that muscle-derived mechanical loading is a principal regulator of bone remodeling and structural strength [32]. Because TBS reflects trabecular texture as a surrogate of bone microarchitecture, studies linking habitual loading, physical activity, and muscle strength to trabecular parameters further support mechanostat-driven adaptation, even though TBS has rarely been used as a primary mechanostat endpoint in controlled trials. A few previous studies have explored these connections. For example, in a cohort of hemodialysis patients, Catalano et al. investigated BMD, TBS, quantitative ultrasound parameters, and handgrip strength, and found that lower handgrip strength was strongly associated with lower TBS and higher estimated 10-year fracture risk [33]. Similarly, in a cross-sectional study of 141 community-dwelling older adults, Seaton et al. showed that upper-body strength predicted higher TBS independently of lumbar spine BMD, whereas gait speed and grip strength were not associated with TBS [34]. In our investigation, areal BMD did not differ between HGS groups, but TBS appeared to provide a more nuanced assessment of bone quality, indicating microarchitectural deterioration despite preserved BMD. Taken together, these data support the findings that muscle strength and habitual loading may be more closely related to trabecular integrity than to areal BMD, and that microarchitectural parameters remain sensitive to mechanical stimuli, lifestyle, and endocrine status [35]. In this context, our results could emphasize HGS may serve as a practical adjunct marker that complements BMD, particularly when combined with TBS.

The novel contribution of this study lies in linking HGS not only with bone quality but also with mental health, resilience, and self-regulation. In our cohort, women with higher HGS had markedly more favorable affective profiles, as anxiety scores were halved compared with those with lower HGS, and severe or extremely severe anxiety was almost absent in the high-HGS group. Similarly, depressive symptoms were significantly less frequent among women with higher HGS. Moreover, HGS scores also correlated inversely with anxiety, depression, and stress levels, supporting a graded association between muscular strength and psychological burden in postmenopausal women. These findings are in line with accumulating evidence that lower muscular strength clusters with poorer mental health in older adults. In a nationwide Korean sample, results have showed that older adults with low HGS had approximately 1.6-fold higher odds of experiencing significant stress compared with those with normal HGS, highlighting stress vulnerability in individuals with reduced strength [19]. Complementing this, a systematic review and meta-analysis by Zasadzka et al., synthesizing 16 studies in adults over the age of 60, found a consistent negative association between HGS and depressive symptoms, indicating that weaker grip strength is associated with more intense depression across diverse community-dwelling older populations [20]. More detailed profiling from the AGUEDA trial in cognitively normal older adults in their early seventies further supports this psychomotor coupling. Bellón et al. showed that greater handgrip strength was moderately associated with higher self-esteem, while higher perceived strength was linked to fewer depressive symptoms [36].

Converging epidemiological and neurobiological evidence supports our observation that preserved muscular strength tends to co-occur with lower psychological distress and more favorable affective functioning in late life. Chronic low-grade systemic inflammation and alterations of hypothalamic-pituitary-adrenal (HPA) axis activity represent shared mechanisms that can simultaneously influence skeletal muscle, bone remodeling, and central mood regulation. Prospective cohort studies in community-dwelling older adults show that higher circulating concentrations of TNF-α and its soluble receptors predict accelerated losses of muscle mass and declines in handgrip strength, underscoring the catabolic impact of persistent inflammation on skeletal muscle and its contribution to sarcopenia [37,38]. In parallel, experimental and clinical work in affective disorders demonstrates that chronic stress-related HPA axis hyperactivity and cortisol excess are associated with hippocampal and prefrontal structural compromise, reduced neurogenesis and synaptic plasticity, and a heightened vulnerability to mood and anxiety symptoms in later life [39,40]. Taken together, these inflammatory and neuroendocrine pathways provide a biologically plausible substrate through which maintained muscular strength and lower inflammatory burden may accompany more resilient affective functioning and reduced psychological distress in older adults.

Resilience and self-regulation are key psychological resources associated with better coping with stress and support adaptive health behaviors across chronic conditions and later life. In our cohort, resilience correlated positively with both TBS and HGS, and inversely with depression, anxiety, and stress, suggesting that more resilient individuals tend to maintain behavioral patterns, such as regular physical activity, balanced nutrition, and adherence to therapeutic regimens, that favor musculoskeletal health [41]. Beyond behavior, resilience has been linked to more favorable neuroendocrine and inflammatory profiles, with evidence that resilient patients show lower levels of psychological distress and may exhibit attenuated cortisol and inflammatory responses to chronic illness [42]. By contrast, psychosocial stress and chronic cortisol elevation can promote bone loss through increased osteoclastogenesis and disruption of bone remodeling homeostasis. Experimental and clinical work indicates that chronic psychological stress and glucocorticoid excess enhance bone resorption, impair osteoblast function, and contribute to osteoporosis risk [43]. Thus, resilience could indirectly preserve bone quality by simultaneously supporting health-promoting behaviors and maintaining a more balanced neuroendocrine milieu.

Self-regulation reflects an individual’s capacity to set goals, monitor behavior, inhibit competing impulses, and adaptively adjust responses when circumstances change or obstacles arise [44]. Within contemporary health psychology models, it is regarded as a core mechanism through which intentions are translated into sustained action, encompassing processes such as planning, self-monitoring, feedback evaluation, and emotion regulation [45]. In the postmenopausal period, when maintaining musculoskeletal health requires long-term adherence to exercise, dietary, and pharmacological recommendations, these self-regulatory capacities become particularly important.

In our cohort, higher self-regulation scores among women with greater muscular strength suggest that psychobehavioral control may be related to consistent engagement in such health-promoting routines. This interpretation is consistent with longitudinal work in community-dwelling older adults showing that executive function and self-regulatory strategies predict better adherence to exercise programs and higher long-term physical activity levels [46]. Engagement in self-regulation behaviors, such as goal-setting, self-monitoring, and planning, enhances self-efficacy and promotes engagement in self-management behaviors [47]. Self-regulation is thus foundational to effective self-management, and interventions that explicitly train goal-setting, action planning, and self-monitoring consistently improve quality of life, symptom control, and healthcare utilization compared with usual care [48,49]. Together, these findings support the view that self-regulation is a modifiable psychological resource that shapes the behavioral pathway linking handgrip strength, bone microarchitecture, and mental health in postmenopausal women.

The association between self-regulation and physical robustness may be bidirectional. On one side, higher self-regulatory capacity is associated with sustained engagement in physical activity and other health behaviors that support muscular strength and bone quality. On the other, better physical function and strength could enhance perceived mastery and self-efficacy, which in turn reinforce motivation and self-regulatory capacity [50]. Recent systematic review and meta-analysis by Xie et al. in older populations show a mutually reinforcing relationship between exercise self-efficacy and physical activity: individuals who are more physically active develop stronger efficacy beliefs, which then promote further activity and better functional outcomes [51]. This type of virtuous cycle, in which good self-regulation promotes better physical function and better physical function reinforces perceived control and psychological resilience, may help explain why physical and psychological well-being often cluster together in older age. This may also partly account for why, in our sample, individuals with higher HGS show better bone quality and fewer psychological difficulties.

From a clinical standpoint, HGS is inexpensive, quick, and highly reproducible, making it a practical adjunct to routine osteoporosis assessment. Used alongside TBS, it may help identify women at higher fracture risk, and particularly those with normal or osteopenic BMD in whom conventional densitometry can underestimate skeletal vulnerability. Moreover, interventions that enhance muscle strength. such as progressive resistance training, adequate protein and vitamin D intake, are likely to yield dual benefits by improving bone quality, preserving physical function, and supporting psychological well-being. The deliberate integration of psychosocial dimensions into musculoskeletal health assessment is therefore especially relevant in postmenopausal care. As shown in our data, resilience and self-regulation independently associate with bone microarchitecture and mental health, suggesting that psychological interventions may usefully complement pharmacologic and lifestyle therapies. Fracture prevention strategies that simultaneously target muscle strength, bone microarchitecture, and psychological resources may ultimately prove more effective than approaches focused on skeletal parameters alone.

This study has several limitations that should be considered when interpreting the findings. First, the cross-sectional design precludes causal inference, as the observed associations between handgrip strength, bone parameters, and psychological measures cannot establish temporality or directionality, and reverse causation is possible. Second, participants were recruited during routine outpatient visits at a single center using convenience sampling, which may introduce selection bias and limits generalizability to broader community-dwelling postmenopausal populations and to women with different sociodemographic and health profiles. Third, confounding is an important concern, as some baseline characteristics differed between handgrip strength groups. Although we adjusted for key covariates in multivariable model, these adjustments may not fully account for group differences or other factors related to both muscle strength and bone or mental health outcomes. Residual and unmeasured confounding therefore remains possible, including from physical activity and sedentary behavior, nutritional status, socioeconomic factors, sleep quality, pain, comorbidity burden, and medication or supplement use that may influence bone metabolism, muscle performance, and psychological well-being. In addition, psychological outcomes were assessed using self-report questionnaires, which are subject to reporting and recall bias and may not fully capture clinical diagnoses. Bone health was assessed using DXA-derived metrics and TBS. Even though widely used, TBS has recognized limitations related to body composition and soft-tissue artifacts. To reduce measurement error, we excluded women with BMI > 37 kg/m^2^, which may further restrict generalizability. Also, handgrip strength was measured at a single visit, and despite standardized procedures, single-time measurements can be influenced by transient factors such as fatigue, pain, and motivation. Finally, the history of mental health problems was assessed using a single self-report item, which may be prone to misclassification and reporting bias. Therefore, we relied primarily on validated symptom measures (DASS-21 subscales) for interpretation.

## 5. Conclusions

In this cross-sectional cohort of postmenopausal women, higher handgrip strength was associated with better trabecular bone microarchitecture, lower psychological distress, and greater resilience and self-regulatory capacity, with both handgrip strength and resilience emerging as independent correlates of TBS. These findings suggest that simple functional testing such as HGS, in combination with TBS and brief psychosocial assessment, could complement conventional fracture risk stratification and help identify women with a more vulnerable musculoskeletal and psychological profile. Future longitudinal and interventional studies should clarify causal pathways between muscular strength, resilience, and skeletal health, ideally testing multimodal programs that combine exercise, nutritional optimization, and psychological or self-management training. In parallel, mechanistic work focusing on neuroendocrine mediators may help define the biological substrate that links physical robustness with mental resilience in postmenopausal women.

## Figures and Tables

**Table 1:** Baseline and anthropometric parameters of investigated population according to handgrip strength in total study population.

Parameter	Lower HGS (*N* = 101)	Higher HGS (*N* = 99)	Total Population (*N* = 200)	*p* *	Effect Size (95% CI) ^§^
Age (years)	69.1 ± 6.9	65.9 ± 6.9	67.5 ± 7.1	0.002	−0.44 (−0.72, −0.16)
Body weight (kg)	70.7 ± 12.2	72.9 ± 11.2	71.8 ± 11.7	0.176	0.19 (−0.09, 0.47)
Body height (cm)	164.8 ± 6.3	167.2 ± 6.4	165.9 ± 6.5	0.009	0.37 (0.09, 0.65)
Body mass index (kg/m^2^)	25.9 ± 3.4	26.1 ± 3.6	26.0 ± 3.5	0.778	0.04 (−0.24, 0.32)
Waist circumference (cm)	89.9 ± 11.1	91.1 ± 10.8	90.5 ± 10.9	0.463	0.10 (−0.17, 0.38)
Hip circumference (cm)	105.2 ± 8.6	105.6 ± 8.0	105.4 ± 8.2	0.767	0.04 (−0.23, 0.32)
Thigh circumference (cm)	55.6 ± 5.6	56.4 ± 5.1	56.0 ± 5.4	0.341	0.13 (−0.14, 0.41)
WHR	0.85 ± 0.05	0.86 ± 0.06	0.86 ± 0.05	0.353	0.13 (−0.15, 0.41)
WHtR	0.55 ± 0.06	0.54 ± 0.06	0.55 ± 0.06	0.742	−0.05 (−0.32, 0.23)
TWR	0.62 ± 0.07	0.63 ± 0.07	0.62 ± 0.07	0.961	0.01 (−0.27, 0.28)
Handgrip strength (kg)	20.1 ± 3.8	28.7 ± 3.2	24.4 ± 5.5	<0.001	2.39 (2.03, 2.76)
Menopause duration (years)	19.9 ± 8.2	17.4 ± 10.1	18.7 ± 9.2	0.051	−0.28 (−0.55, 0.00)
Smoking (active)	17 (16.8)	28 (28.3)	45 (22.5)	0.053 ^†^	
Bone-effecting NCD ^‡^	43 (42.6)	44 (44.4)	87 (43.5)	0.790 ^†^	
Fragility fracture (yes)	26 (25.7)	9 (9.1)	35 (17.5)	0.002 ^†^	
Bone loss therapy					
None	39 (38.6)	42 (42.4)	81 (40.5)	0.899 ^†^	
Vitamin D/calcium	44 (43.6)	38 (38.4)	82 (41.0)		
Bisphosphonates	8 (7.9)	9 (9.1)	17 (8.5)		
Monoclonal antibody	10 (9.9)	10 (10.1)	20 (10.0)		

Data are presented as mean ± SD or whole number (%). WHR—waist-to-hip ratio; WHtR—waist-to-height ratio; TWR—thigh-to-waist ratio; NCD—non-communicable diseases; HGS—handgrip strength. * *t*-test for independent samples. ^†^ chi-squared test. ^‡^ rheumatoid arthritis/malignant disease/diabetes/endocrinopathies/glucocorticoid therapy. ^§^ Hedges’ g effect size measure.

**Table 2:** Bone health parameters and 10-year fracture risk calculated with FRAX tool in study population according to handgrip strength.

Parameter	Lower HGS (*N* = 101)	Higher HGS (*N* = 99)	Total Population (*N* = 200)	*p* *
Lumbar L_1_-L_4_ BMD (g/cm^2^)	0.85 (0.77; 0.98)	0.83 (0.75; 0.95)	0.83 (0.76; 0.96)	0.278
Lumbar L_1_-L_4_ T-score	−1.85 (−2.5; −0.47)	−2.0 (−2.7; −0.9)	−1.9 (−2.6; −0.7)	0.193
Lumbar L_1_-L_4_ Z-score	0.3 (−0.52; 1.52)	0.0 (−0.9; 0.8)	0.1 (−0.8; 1.35)	0.025
Total hip BMD (g/cm^2^)	0.77 (0.68; 0.82)	0.75 (0.69; 0.82)	0.76 (0.68; 0.82)	0.565
Total hip T-score	−1.4 (−2.1; −0.9)	−1.6 (−2.1; −1.0)	−1.5 (−2.1; −1.0)	0.548
Total hip Z-score	0.1 (−0.6; 0.7)	−0.3 (−1.0; 0.45)	−0.1 (−0.9; 0.6)	0.067
Femur neck BMD (g/cm^2^)	0.66 (0.61; 0.74)	0.67 (0.61; 0.76)	0.67 (0.61; 0.75)	0.947
Femur neck T-score	−1.6 (−2.1; −0.9)	−1.6 (−2.1; −0.8)	−1.6 (−2.1; −0.9)	0.929
Femur neck Z-score	0.2 (−0.5; 0.92)	−0.1 (0.5; 0.8)	0.1 (−0.5; 0.9)	0.224
MOF risk (%) ^†^	11.0 (8.7; 16.2)	9.9 (7.3; 15.0)	10.0 (7.6; 16.0)	0.163
Hip fracture risk (%) ^†^	1.9 (0.8; 3.8)	1.4 (0.5; 2.97)	1.7 (0.7; 3.5)	0.114
MOF—TBS adjusted risk (%) ^†^	13.0 (9.9; 17.2)	10.0 (7.7; 15.0)	12.0 (8.4; 16.0)	0.015
Hip fracture—TBS adjusted risk (%) ^†^	2.4 (1.1; 4.1)	1.5 (0.52; 3.37)	2.0 (0.8; 3.8)	0.017
Bone mineral density status				
Normal	13 (12.9)	13 (13.1)	26 (13.0)	0.876 ^†^
Osteopenia	53 (52.5)	50 (50.5)	103 (51.5)	
Osteoporosis	35 (34.7)	36 (36.4)	71 (35.5)	

Data are presented as median (IQR) or whole number (%). FRAX—Fracture Risk Assessment Tool; HGS—Handgrip Strength; TBS—Trabecular Bone Score; MOF—Major Osteoporotic Fracture; BMD—Bone Mineral Density. * Mann–Whitney U test. ^†^ chi-squared test.

**Table 3:** Mental health parameters in study population according to handgrip strength.

Parameter	Lower HGS (*N* = 101)	Higher HGS (*N* = 99)	Total Population (*N* = 200)	*p* *
DASS-A score	4.0 (2.0; 10.0)	2.0 (2.0; 4.0)	4.0 (2.0; 8.0)	0.011
DASS-A categories				
Normal	62 (61.4)	87 (87.8)	149 (74.5)	<0.001 ^†^
Mild/Moderate	25 (24.8)	11 (11.1)	36 (18.0)	
Severe/Extremely severe	14 (13.9)	1 (1.0)	15 (7.5)	
DASS-D score	2.0 (0.0; 6.0)	2.0 (0.0; 4.0)	2.0 (0.0; 4.0)	0.013
DASS-D categories				
Normal	82 (81.2)	94 (94.9)	176 (88.0)	0.009 ^†^
Mild/Moderate	17 (16.8)	5 (5.1)	5 (5.1)	
Severe/Extremely severe	2 (2.0)	0 (0.0)	0 (0.0)	
DASS-S score	6.0 (2.0; 14.0)	6.0 (2.0; 10.0)	6.0 (2.0; 12.0)	0.136
DASS-S categories				
Normal	77 (76.2)	90 (90.9)	167 (83.5)	0.019 ^†^
Mild/Moderate	20 (19.8)	8 (8.1)	8 (8.1)	
Severe/Extremely severe	4 (4.0)	1 (1.0)	5 (2.5)	
Self-assessed MHD history (yes)	26 (25.7)	14 (14.1)	40 (20.0)	0.041 ^†^

Data are presented as median (IQR) or whole number (%). DASS-A—Depression, Anxiety, and Stress Scale—Anxiety; DASS-D—Depression, Anxiety, and Stress Scale—Depression; DASS-S—Depression, Anxiety, and Stress Scale—Stress; HGS—Handgrip Strength; MHD—Mental Health Disorder. * Mann–Whitney U test. ^†^ chi-squared test.

**Table 4:** Correlation of handgrip strength, BRS, and SSRQ score with relevant parameters in whole study population (***N*** = 200).

Parameter r (*p*) *	HGS (kg)	BRS Score ^†^	SSRQ Score
Age (years)	−0.227 (0.001)	−0.108 (0.129)	−0.125 (0.078)
Body mass index (kg/m^2^)	0.075 (0.292)	−0.070 (0.325)	−0.111 (0.117)
Waist circumference (cm)	0.109 (0.126)	−0.060 (0.396)	−0.111 (0.117)
WHR	0.058 (0.416)	−0.023 (0.749)	−0.132 (0.062)
WHtR	−0.061 (0.389)	−0.100 (0.158)	−0.162 (0.022)
TWR	0.010 (0.887)	0.116 (0.101)	0.166 (0.019)
Menopause duration (years)	−0.136 (0.054)	−0.059 (0.403)	−0.116 (0.103)
Lumbar L1-L4 BMD (g/cm^2^)	−0.073 (0.305)	−0.018 (0.798)	−0.107 (0.131)
Total hip BMD (g/cm^2^)	−0.071 (0.317)	0.063 (0.372)	−0.095 (0.179)
Femur neck BMD (g/cm^2^)	0.004 (0.951)	0.032 (0.652)	−0.079 (0.268)
TBS	0.249 (<0.001)	0.206 (0.003)	0.036 (0.613)
MOF risk (%) ^†^	−0.098 (0.168)	−0.025 (0.730)	−0.035 (0.624)
Hip fracture risk (%) ^†^	−0.119 (0.093)	−0.051 (0.475)	−0.041 (0.567)
MOF—TBS adjusted risk (%) ^†^	−0.185 (0.008)	−0.070 (0.322)	−0.049 (0.487)
Hip fracture—TBS adjusted risk (%) ^†^	−0.175 (0.013)	−0.075 (0.293)	−0.042 (0.554)
DASS-A score ^†^	−0.255 (<0.001)	−0.248 (<0.001)	−0.297 (<0.001)
DASS-D score ^†^	−0.248 (<0.001)	−0.331 (<0.001)	−0.393 (<0.001)
DASS-S score ^†^	−0.200 (0.005)	−0.360 (<0.001)	−0.395 (<0.001)

BMI—Body Mass Index; BMD—Bone Mineral Density; HGS—Handgrip Strength; BRS—Brief Resilience Scale; SSRQ—Short Self-Regulation Questionnaire; DASS-A—Depression, Anxiety, and Stress Scale—Anxiety; DASS-D—Depression, Anxiety, and Stress Scale—Depression; DASS-S—Depression, Anxiety, and Stress Scale—Stress; WHR—waist-to-hip ratio; WHtR—waist-to-height ratio; TWR—thigh-to-waist ratio; TBS—Trabecular Bone Score; MOF—Major Osteoporotic Fracture. * Pearson’s correlation coefficient. ^†^ Spearman’s correlation coefficient.

## Data Availability

The data that were presented in this study are available on request from the corresponding author. The data are not accessible to the public due to ethical constraints.
